# Periprosthetic fractures around total knee arthroplasty

**DOI:** 10.1308/10.1308/003588412X13171221592537

**Published:** 2012-07

**Authors:** SS Sarmah, S Patel, G Reading, M El-Husseiny, S Douglas, FS Haddad

**Affiliations:** University College Hospital,London,UK

**Keywords:** Knee replacement arthroplasty, Periprosthetic fractures, Trauma

## Abstract

**INTRODUCTION:**

The number of total knee arthroplasties performed continues to rise annually and it would be expected that complications, which include periprosthetic fractures, will also therefore become more commonplace. This article reviews the current literature regarding this injury and identifies the treatment principles that enable patients to regain optimal function.

**METHODS:**

A comprehensive search of the Pubmed and Embase™ databases was performed to identify relevant articles. Keywords and MeSH (Medical Subject Headings) terms included in the search strategy were ‘periprosthetic fracture(s)’, ‘femur’, ‘tibia’, ‘patella(r)’, ‘complication(s)’, ‘failure(s)’, ‘risk(s)’, ‘prevalence’, ‘incidence’, ‘epidemiology’ and ‘classification(s)’. The search was limited to all articles published in English and reference lists from the original articles were reviewed to identify pertinent articles to include in this review. A total number of 43 studies were identified.

**RESULTS:**

Common treatment aims have been identified when managing patients with a periprosthetic fracture around total knee arthoplasty. The main criterion that determines which option to choose is the degree of remaining bone stock and the amount of fracture displacement.

**CONCLUSIONS:**

Treatment of a periprosthetic fracture around total knee arthroplasty will either be non-operative, osteosynthesis or revision arthroplasty. It is imperative that a suitable option is chosen and based on the published literature, pathways are outlined to aid the surgeon.

Increasing demand for high end activity in the middle to late age, improved life expectancy and obesity are all factors suggested for the continued rise in total knee arthroplasties (TKAs) performed annually. Concurrently, the number of periprosthetic fractures (PPFs) witnessed is also rising^1^ and, while the main goal of achieving a stable, painless joint without gross malalignment is possible in the majority of patients following this injury,^2^ it remains a challenging clinical scenario for both surgeon and patient. The purpose of this review was to summarise the epidemiology, aetiology and classification of this injury before outlining the principles of management and offering treatment pathways.

## Epidemiology and aetiology

Data published in 2011 from a Scottish national database recording 47,000 TKAs over 11 years estimate the risk of PPF to be 0.6% in the first five years after primary TKA and 1.7% after revision TKA.^3^ In particular, patients aged ≥70 years were 1.6 times more likely to have a fracture than younger patients and women were overall 2.3 times more likely to suffer a fracture than men.

**Figure 1:**
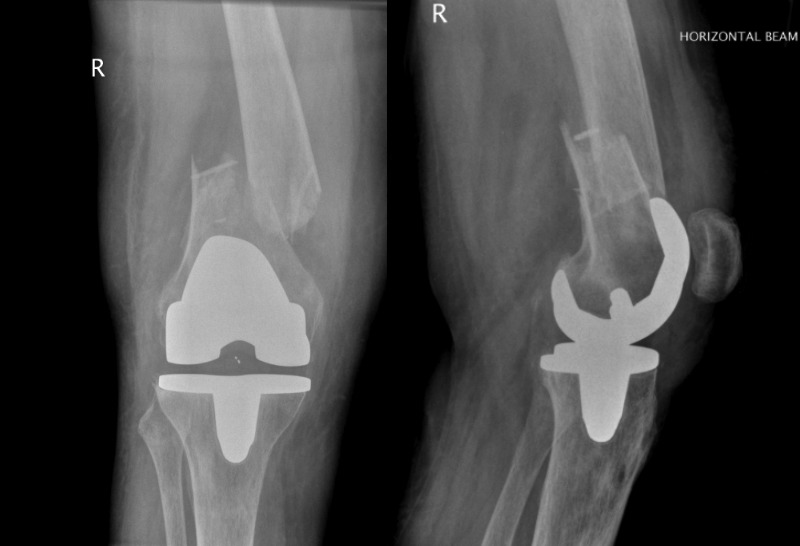
Anteroposterior and lateral radiographs sustaining a Lewis and Rorabeck type II periprosthetic fracture

The location of the fracture invariably alters management and, to that end, the majority of PPFs occur in the supracondylar region of the femur; the incidence is reported as being between 0.3% and 2.5% of all TKAs.^4–6^ The patella is the next most frequently affected site of PPF with an incidence of 0.68–1.19%^7,8^ with a tendency to occur more frequently post-operatively than intra-operatively.^9,10^ These fractures are 2.5 times more likely to occur in men than in women,^7^ thereby bucking the overall trend. While associated with resurfacing, they are also witnessed in the non-resurfaced patella.^9,11^ The male predilection is thought to be secondary to their higher activity level and body weight leading to greater extensor mechanism forces and patellofemoral stresses.^7^ PPFs of the tibia, meanwhile, are rare, occurring in just 0.1% of cases intra-operatively and 0.4% post-operatively.^12^

No single aetiological factor can be attributed to all PPFs since they may occur in any patient who has undergone a TKA. However, a number of conditions or incidents are associated that can be broadly divided into intrinsic and extrinsic factors (Table 1). It is beyond the scope of this paper to discuss all but the most important in further detail.

**Table 1:** Predisposing factors associated with periprosthetic fractures around total knee arthroplasties

**Intrinsic factors** Demographic > Age ≥70 years > Female sex **Activity** > Trauma > High activity level **Medical disorders** > Decreased bone mineral density > Chronic steroid usage > Rheumatoid arthritis > Epilepsy > Parkinsonism > Myasthenia gravis > Poliomyelitis > Cerebral palsy
**Extrinsic factors** Femur > Anterior femoral notching > Component malpositioning > Poorly reamed bone > Stress shielding > Box cut for posterior stabilised implants **Patella** > Excessive bony resection > Central peg > Press-fit implants > Lateral release > Fat pad excision > Maltracking > Cement heat necrosis **Tibia** > Malalignment > Osteolysis > Sclerotic subchondral bone > Intramedullary referencing > Tibial tubercle osteotomy

Unsurprisingly, trauma is a cause of PPF but this need not be high energy episodes since they do occur following low energy incidents such as a simple fall, in which axial and torsional forces combine.^13^ This is especially the case for patients whose mechanical strength of bone is compromised as is witnessed in chronic conditions such as osteoporosis and steroid usage. Compounding these particular patients is the knowledge that poor bone quality may compromise fixation and lead to complications including non-union, angular deformity, implant migration and limb shortening.

The inclusion of stress risers is a suggested cause of PPF of the femur, in particular notching of the anterior cortex. Biomechanical studies have demonstrated that notches greater than 3mm deep, sharper notches and a notch close to the femoral prosthesis will all influence the local stress concentration, potentially reducing torsional bone strength by 30–40% and flexural strength by 18%^14,15^ while decreasing bone mineral density will further potentiate the risk of PPF.^16^

Clinically, however, the presence of a notch has not been proven conclusively as an independent factor in the more recently published data on this subject. In 2005 Ritter *et al* found no association between notching and PPF.^17^ They reported that 30% of patients in their series of over 1,000 knees with a mean 5-year follow-up duration had radiographic evidence of notching with a mean depth of 2.5mm and increasing up to 10mm (0.2% of knees). Of this cohort, only two supracondylar femoral fractures occurred and neither of these femurs were notched.

In 2009 Gujarathi *et al* presented data on 200 TKAs with a mean follow-up duration of 9 years and discovered that of the 3 patients who had sustained a supracondylar fracture, only 1 femur was notched.^18^ It is difficult to understand the disparity between the biomechanical and clinical data although the overall low incidence of PPF may not allow for a causal relationship to be identified unless larger scale or national series are investigated.

Poor bone stock and devascularisation (with subsequent osteonecrosis) are both implicated as the main underlying factors predisposing to patellar fracture outside of traumatic episodes since they increase fatigue stresses directly and indirectly respectively. On that basis, it is recommended that a new patellar component should not be implanted when the remaining thickness of the patellar is less than 10mm^19^ while frequently performed techniques such as arthrotomy, patellar eversion and lateral release all interrupt the osseous blood supply of the patella.^20,21^ To that end, using the subvastus approach and lateral retraction of the patella may lessen the likelihood of vascular compromise and possibly PPF although this is remains unproven.

PPFs of the tibia may occur intra-operatively or post-operatively. Intra-operative fractures happen during component removal, bone retraction, trial reduction and preparation for insertion of a stemmed tibial component.^22^ Post-operative fractures are associated with malalignment, component loosening and osteolysis by means of increasing stress on the tibia and altering its structural integrity.

## Classification and treatment

Multiple classification systems exist to describe the patterns of PPF that occur. They are broadly described according to site, displacement, component loosening and remaining bone stock.

### Femur

The classification described by Lewis and Rorabeck^23^ is long used and divides the injury into three types:

> Type I: undisplaced fracture and prosthesis is well fixed
> Type II: displaced fracture and prosthesis is well fixed
> Type III: prosthesis is loose, fracture may be displaced or undisplaced

Based on this classification, the original authors advocated non-operative treatment for type I fractures, either closed reduction and fixation with an intramedullary nail or open reduction and internal fixation with a plate for type II fractures, and revision of the prosthesis using long stemmed revision implants or structural allograft depending on the bone stock available for type III fractures.

An alternative classification system was proposed by Kim *et al*,^2^ in which injuries were divided into four categories based on whether the fracture was reducible, whether there was sufficient bone stock in the distal fragment, and whether the component was well positioned and well fixed. The addition of a new subtype allowed for inclusion of a new treatment option, namely distal femoral replacement (Table 2). Non-operative treatment is therefore reserved for undisplaced, stable fractures with a well fixed implant or for high risk patients^24^ and it is achieved by cast immobilisation either with or without prior skeletal traction.^5,25,26^

**Table 2:** Kim *et al’*s classification of supracondylar femoral fractures^2^

Type	Reducible fracture	Bone stock in distal fragment	Well positioned and well fixed component	Management
IA	Yes	Good	Yes	Conservative
IB	No	Good	Yes	Surgical
II	–	Good	No	Revision with long stem
III	–	Poor	No	Prosthetic replacement

When considering osteosynthesis, it is imperative that the implant is well fixed since revision arthroplasty needs to otherwise be considered. The outcomes of selected case series in which operative and non-operative treatment have been used are summarised in Table 3. Selecting the appropriate stabilisation device depends on several factors:

> location of fracture relative to component (ie proximal or distal)
> displacement of fracture and comminution
> fracture pattern
> presence of other implants in the proximal femur (eg hip arthroplasty prosthesis)

**Table 3:** Studies describing the number of patients, methods and outcomes of treatment for supracondylar femoral periprosthetic fractures

Study group	Non-operative treatment	Non-operative outcome	Operative treatment	Operative outcome
Sisto *et al*^41^	4 – cast, 8 – traction	11 U, 1 NU	3 RIF	3 U
Merkel and Johnson^5^	26 – cast/brace	17 U, 2 MU, 4 NU, 2 LC, 1 EL	5 RIF, 3 REF	1 Excellent, 2 Good, 3 Satisfactory, 1 NU, 1 AKA
Culp *et al*^42^	30 – cast/brace	17 U, 7 MU, 6 NU	31 RIF	25 U, 3 MU, 1 NU
Moran *et al*^43^	14 – cast/brace	5 U, 9 NU	15 RIF	10 U, 2 MU, 3 NU
Platzer *et al*^24^	3 – cast, 1 – traction	Not stated	30 RIF, 3 RA	3 NU in plated group, 1 NU in nailed group

NU = non-union; RIF = reduction and internal fixation; U = union; MU = malunion; LC = loose component; EL = extensor lag; REF = reduction and external fixation; AKA = above knee amputation; RA = revision arthroplasty

Broadly speaking, stabilisation devices are intra or extramedullary. Intramedullary nails are the best example of the former and, where a cruciate retaining device has been used at the index procedure, the nail can be introduced in a retrograde manner. They provide a relative risk reduction of 87% for developing a non-union and 70% for requiring revision surgery when compared with non-locking plates^27^ but are best avoided when an ipsilateral hip prosthesis is in situ due to the creation of a stress riser between the two components.

The advent of locking plates has provided an extramedullary option that is particularly useful when poor bone stock is present.^28^ Lower rates of complications (12% vs 42%), malunion (20% vs 47%) and non-union (0% vs 16%) have been reported when using them compared with non-locking plates and intramedullary fixation.^29^ However, the presence of medial comminution can increase the risk of failure if only lateral plate stabilisation is used and thus while locking plates are useful in most cases, there is an argument for specifically using an intramedullary nail here.^30^

Although infrequently used, external fixators also have satisfactory outcomes in high risk surgical patients.^31–33^

Revision of the femoral stem with a cemented long stem prosthesis is a prerequisite when a loose implant is encountered.^34^ The potential for complications is high since a greater soft tissue dissection is required and this further devascularises the bone, thereby reducing the likelihood of osseous union. Poor bone stock further complicates a loose prosthesis as there may be insufficient support for new hardware. The options available include reconstruction of the distal femur using an allograft or alternatively distal femoral replacement. Both methods have shown improved overall function although high rates of complication and especially infection mean that they are best reserved for patients where alternative treatments are not possible_ENREF_34.^35–38^

### Patella

The most widely used system to classify patellar fractures is that proposed by Ortiguera and Berry^7^ in which the defining parameters include integrity of the extensor mechanism, fixation status of the patellar component and quality of residual bone stock. There are four types:

> Type I; well fixed prosthesis with intact extensor mechanism
> Type II: well fixed prosthesis with disrupted extensor mechanism
> Type IIIa: loose prosthesis with reasonable bone stock
> Type IIIb: loose prosthesis with poor bone stock (<10mm thick or marked comminution)

An additional note is that a loose prosthesis coexisting with a disrupted extensor mechanism is classed as a type II fracture.

Type I fractures are amenable to non-operative treatment with the senior author preferring initial immobilisation in full extension before commencing a graduated increase in motion once radiographic signs of union occur. Non-operative treatment of this type of fracture has good outcomes in over 96% of patients, which includes those with non-union where a fibrous bar allows function to be maintained.^7,39^

In contrast, operative treatment has universally poor results and it has been argued that non-operative treatment of displaced fractures with substantial associated extensor lag is acceptable as long as fixation of the patellar component is maintained.^22^ This argument is supported by information garnered from a grouped analysis that discovered a non-union rate of 92% after internal fixation with tension-band technique or cerclage wire leading to poor results in the majority of cases.^8^ In the absence of a randomised trial for displaced fractures, however, the majority of published studies still advocate operative treatment for displaced fractures in a bid to maximise potential function.

In a similar manner to PPFs of the femur, the residual bone stock and fixation of the patellar component are of paramount importance when deciding what surgical option to take in tackling displaced PPFs of the patella. A minimum thickness of 10mm is required when considering revision arthroplasty although, if the bone stock is deemed insufficient, resection arthroplasty is a better option even if it causes reduced quadriceps strength and consequent persistent extensor lag.^40^

**Figure 2:**
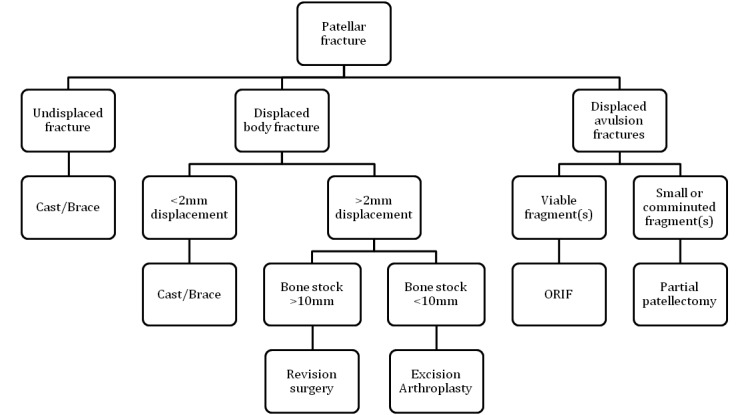
Treatment algorithm for the management of patellar periprosthetic fractures

Injuries occurring at either the proximal or distal pole should be managed in the same manner as a virgin knee in that the fracture ends should be reapproximated where possible and, if required, the repair augmented with either autograft or allograft. Where the remaining bone is either too small or too comminuted to support a repair, a partial patellectomy can be performed. Using the information presented thus far, the senior author’s preferred method of treatment is summarised in Figure 2.

### Tibia

**Figure 3:**
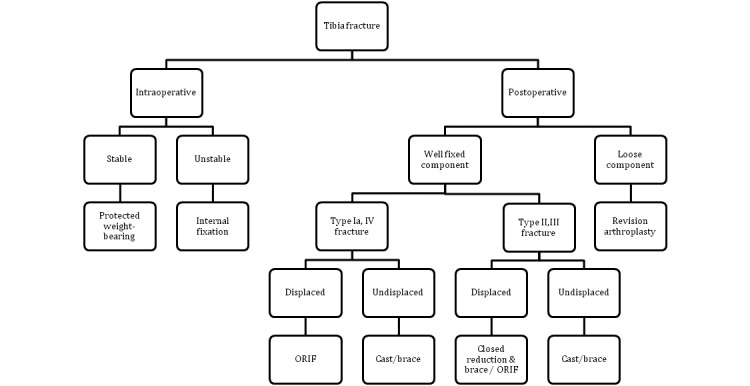
Treatment algorithm for the management of tibial periprosthetic fractures

The Mayo classification described by Felix *et al* in 1997^12^ is the most widely used system to classify tibial PPFs. Information on the location of the fracture, the stability of the implant and whether the fracture occurred intra-operatively or post-operatively is included, and treatment can be guided accordingly (Fig 3).

Type I fractures consist of a depression or split of the tibial plateau and extend to the interface of the implant, type II fractures occur adjacent to the stem, type III fractures are diaphyseal fractures occurring distal to the prosthesis and type IV fractures are avulsion injuries of the tibial tubercle. Types I–III are further subtyped A, B or C depending on whether the prosthesis is well fixed, loose or whether the fracture occurred intra-operatively respectively.

As with other PPFs of the femur and patella, non-operative treatment is advocated for stable, undisplaced fractures, reduction is required for displaced injuries and revision arthroplasty is needed if a loose implant is encountered. There are further management guidelines for types IIIB, IC and IIC fractures. Namely, when a type IIIB fracture occurs, the site of injury is away from the prosthesis and therefore revision arthroplasty may be best undertaken at a later date once the fracture has been allowed to heal. Furthermore, surgery for types IC and IIC should be revised at the index procedure to ensure a long stemmed implant is used that traverses the fracture site to provide additional stability.

## Conclusions

PPFs around TKA are rare injuries but can be complex to treat. The challenges faced include poor bone stock and a diminished healing capacity with poor biological and physiological reserve in the elderly or chronically unwell. Non-operative management is usually reserved for patellar fractures, undisplaced fractures with stable prostheses and high risk patients is whom surgery would be life threatening. Non-union is still witnessed today and is attributed to the disruption of the endosteal blood supply that occurs with the injury or during surgical dissection. In spite of this, it should be remembered that surgery can lessen the complications associated with prolonged immobilisation. The treatment pathways offered in this article provide an evidence-based approach to management and are there to aid the surgeon in decision making.
